# Algorithm sensitivity analysis and parameter tuning for tissue image segmentation pipelines

**DOI:** 10.1093/bioinformatics/btw749

**Published:** 2016-12-30

**Authors:** George Teodoro, Tahsin M Kurç, Luís F R Taveira, Alba C M A Melo, Yi Gao, Jun Kong, Joel H Saltz

**Affiliations:** 1 Department of Computer Science, University of Brasília, Brasília, Brazil; 2 Biomedical Informatics Department, Emory University, Atlanta, GA, USA; 3 Biomedical Informatics Department, Stony Brook University, Stony Brook, NY, USA; 4 Scientific Data Group, Oak Ridge National Laboratory, Oak Ridge, TN, USA

## Abstract

**Motivation:**

Sensitivity analysis and parameter tuning are important processes in large-scale image analysis. They are very costly because the image analysis workflows are required to be executed several times to systematically correlate output variations with parameter changes or to tune parameters. An integrated solution with minimum user interaction that uses effective methodologies and high performance computing is required to scale these studies to large imaging datasets and expensive analysis workflows.

**Results:**

The experiments with two segmentation workflows show that the proposed approach can (i) quickly identify and prune parameters that are non-influential; (ii) search a small fraction (about 100 points) of the parameter search space with billions to trillions of points and improve the quality of segmentation results (Dice and Jaccard metrics) by as much as 1.42× compared to the results from the default parameters; (iii) attain good scalability on a high performance cluster with several effective optimizations.

**Conclusions:**

Our work demonstrates the feasibility of performing sensitivity analyses, parameter studies and auto-tuning with large datasets. The proposed framework can enable the quantification of error estimations and output variations in image segmentation pipelines.

**Availability and Implementation:**

Source code: https://github.com/SBU-BMI/region-templates/.

**Supplementary information:**

Supplementary data are available at *Bioinformatics* online.

## 1 Introduction

Whole slide tissue images (WSIs) obtained from tissue specimens provide a means to study disease morphology at the sub-cellular scale. Several algorithm, computation and data challenges, however, have to be overcome in order to facilitate studies with large datasets of WSIs. In this work we target challenges that stem from the fact that most image analysis workflows are sensitive to variations in input parameters. A workflow optimized for a group of images may not perform well for another set of images. It is, therefore, important to (1) quantify the impact of input parameters on analysis output and (2) adjust parameters to produce more accurate analysis results. We call (1) and (2) collectively a *parameter study*. We refer to part (1) as the *algorithm sensitivity analysis (SA) process* and define it as the process of comparing results from multiple analyses of a dataset using variations of an analysis workflow (e.g. different input parameters or different algorithms) and quantifying differences in the results. Part (2) is an extension of SA and we refer to it as the *parameter tuning process*. We are interested in parameter auto-tuning in which the parameter space of an analysis workflow is searched automatically by generating analysis results from a set of parameters, comparing the analysis results with *ground truth*, and repeating the process to find a set of parameters that produces the most accurate results as measured by a comparison metric.

A parameter study with a large set of WSIs can be a challenging and computationally expensive task. Consider a workflow of normalization and segmentation steps—this type of workflow is our focus in this paper, because segmentation is a crucial step in extracting salient morphology information from images and consists of several complex and parameterized data transformation operations. Processing a single WSI through this workflow can take hours on a single CPU. In addition, SA may require the evaluation of hundreds or thousands of parameter combinations because of the staggeringly high number of possible parameter values. Table 1 shows the list of parameters and their value ranges for the segmentation step in two analysis workflows used in our experimental evaluation. The first workflow uses a watershed based segmentation step (Kong *et al.*, 2013), while the other employs level set and mean shift clustering methods (Gao *et al.*, 2016). The large number of possible parameter values can lead to a large number of runs and high volumes of data to be handled and processed. For instance, one of our SA experiments involved 2000 runs of the watershed segmentation pipeline on 55 WSIs. The execution of the experiment on a distributed memory machine with 128 nodes took 42.9 h; and during the experiment, a total of 820 Terabytes of data were produced and processed. In studies at this scale it is imperative to utilize high performance computing systems efficiently in order to speed up the parameter study processes.

**Table 1:** Parameters their value ranges for two example workflows

Parameter	Description	Range value
(a) Parameters of the Watershed based segmentation workflow. The parameter search space contains about 21 trillion parameter points		
B/G/R	Background detection thresholds	[210, 220,…, 240]
T1/T2	Red blood cell thresholds	[2.5, 3.0,…, 7.5]
G1/G2	Thresholds to identify	[5, 10,…, 80]
	candidate nuclei	[2, 4,…, 40]
MinSize	Area threshold of candidate nuclei	[2, 4,…, 40]
MaxSize	Area threshold of candidate nuclei	[900,., 1500]
MinSizePl	Area threshold before watershed	[5, 10,…, 80]
MinSizeSeg	Area threshold from final segmentation	[2, 4,…, 40]
MaxSizeSeg	Area threshold from final segmentation	[900,., 1500]
FillHoles	propagation neighborhood	[4-conn, 8-conn]
MorphRecon	propagation neighborhood	[4-conn, 8-conn]
Watershed	propagation neighborhood	[4-conn, 8-conn]
(b) Parameters of the Level Set based segmentation workflow. The parameter search space contains about 2.8 billion parameter points		
OTSU	OTSU threshold value	[0.3, 0.4,…, 1.3]
Curvature Weight	Curvature weight (CW) for level-set	[0.0, 0.05,…, 1.0]
MinSize	Minimum object size	[1, 2,…, 20]
MaxSize	Maximum object size	[50, 55,…, 400]
MsKernel	Radius in Mean-Shift calculation	[5, 6,…, 30]
LevetSetIt	Number of iterations of	[5, 6,…, 150]
	the level set computation	

Approaches for parameter space search and parameter optimization have been developed and successfully applied in several application domains (Campolongo *et al.*, 2007; Jones, 2001; Morris, 1991; Rios and Sahinidis, 2013; Saltelli *et al.*, 2004; Sareni and Krähenbühl, 1998; Snoek *et al.*, 2012; Tabatabaee *et al.*, 2005; Weirs *et al.*, 2012). One of the main contributions of this work is the adaptation of these methods and demonstration of their utility in the context of large-scale whole slide tissue image analysis. This is accomplished by integrating the parameter search and auto-tuning methods into a software framework that employs novel high-performance computing (HPC) techniques to address the data and computational challenges. Our contributions can be summarized as follows.

- An approach for efficient execution of sensitivity analysis for WSI segmentation workflows. Our approach implements a global SA process, which examines output sensitivity from the perspective of a range of input parameter variations (Iooss and Lemaitre, 2015). A key consideration is to reduce the number of parameter evaluations and provide relevant information about the impact of input parameters on analysis results. Our approach adapts and incorporates two types of methods (Campolongo *et al.*, 2007; Morris, 1991; Sobol, 2001; Weirs *et al.*, 2012) for use in WSI analysis to accomplish this: (1) methods to efficiently perform an initial and quick *screening* of non-influential input parameters (i.e. those parameters that do not contribute significantly to variations in output) and remove them from further consideration; and (2) methods that compute *measures of importance* or quantitative sensitivity indexes. The combination of these methods enables the application of sensitivity analysis with large datasets of WSIs and for segmentation workflows with large parameter spaces.
- A systematic experimental evaluation of multiple optimization algorithms for automatically tuning input parameters in segmentation workflows. Previous work on automated parameter estimation optimization in image segmentation has employed techniques for specific segmentation models (Kumar and Hebert, 2003; McIntosh and Hamarneh, 2007; Schultz and Kindlmann, 2013; Szummer *et al.*, 2008). The Tuner system (Torsney-Weir *et al.*, 2011) targets general segmentation algorithms and uses statistical models from sampling runs to explore the parameter space. In our work, we treat the segmentation algorithm as a black-box and employ efficient optimization algorithms that can quickly converge to desired results. Our results show that the output of a segmentation workflow can be significantly improved with the automated tuning approaches. In our experiments, the quality of the segmentation results was improved, as measured by the Dice (1945) and Jaccard metrics, by as much as 42% compared with the results obtained with the default workflow parameters.
- High performance computing methods to address the computation and data challenges in parameter studies. Our work provides several runtime optimizations to accelerate parameter evaluation runs on distributed-memory parallel machines. These methods are integrated in a runtime system, called Region Templates, we developed in an earlier work (Teodoro *et al.*, 2014). The Region Templates system is designed to address the processing and data management challenges of image analysis pipelines on distributed memory systems with hybrid multi-core CPU and co-processors. The work described in this paper adds new optimizations for parameter studies: (1) efficient data movement and staging, data-aware assignment of stages and operations in an analysis workflow to optimize repeated workflow executions with different parameters; and (2) simultaneous parameter evaluation to eliminate common computations in the execution of an analysis workflow with multiple parameter sets. The combined use of these optimizations is crucial to enabling large-scale studies.

## 2 Methods

**Fig. 1.**
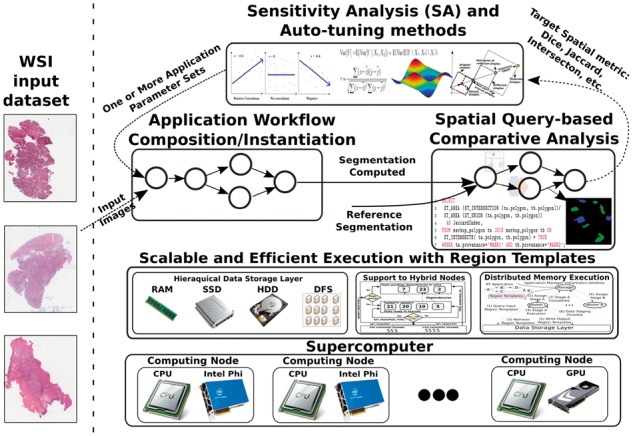
The parameter study framework. A parameter study process (SA or auto-tuning) is selected by an investigator. The analysis workflow is executed on a parallel machine multiple times while input parameters are systematically varied. The analysis results are compared to a set of reference results to compute a new set of parameters. This iterative process continues until the process has converged in the case of the parameter tuning process or collected enough data in the case of the sensitivity analysis process (Color version of this figure is available at *Bioinformatics* online.)

Our parameter study framework is illustrated in Figure 1. An investigator specifies a set of input images, an image analysis workflow, the value ranges of input parameters for the image analysis workflow, and the metric of interest (e.g. Dice) for comparison of analysis results. The image analysis workflow is executed on a high performance machine, while the input parameters are systematically varied by the framework.

The sensitivity analysis process is carried out in two phases. During these phases, multiple runs of the image analysis workflow are executed until the required set of parameters has been covered. In the first phase, a set of runs are executed and *screening* methods are called. The screening methods are used to determine which parameters of an analysis workflow have little impact on output variability—such parameters are called non-influential parameters (Section 2.1). The screening step is used as a filtering step with a large number of parameter values, before the more costly second phase is executed. At the end of the first phase, the investigator may remove some of the parameters from further consideration and fix their values in the second phase. The second phase computes importance measures for selected parameters (Section 2.2). This phase looks at the monotonicity and linearity of an analysis workflow’s output and correlates variance in the output with the input parameters and their first-order and higher-order effects. The results from the sensitivity analysis process are statistics that quantify variance in the analysis results as well as measures such as sensitivity indices that indicate the amount of variance in the analysis results that can be attributed to individual parameters or combinations of parameters (Saltelli, 2002; Sobol, 2001).

The parameter auto-tuning process calibrates the input parameters to generate more accurate results and requires a reference dataset (see Section 2.3). The reference dataset for tuning a segmentation pipeline can be, for example, a set of segmentation results generated by human experts. In the auto-tuning process, image analysis results (i.e. sets of segmented objects in our case) generated from a set of input parameter values are compared to the reference dataset. The comparison step computes an *error estimate* based on a metric, such as Dice, and feeds it to a search optimization method to generate another set of parameter values. This iterative process continues until a maximum number of iterations is reached or when the error estimate is below a threshold.

### 2.1 Methods to screen input parameters

Our implementation employs a commonly used screening method, called Morris One-At-A-Time (MOAT) design (Morris, 1991). This screening method perturbs each input parameter in a discretized parameter space while fixing the other input parameters. The *k*-dimensional input space (for *k* parameters) is partitioned uniformly in *p* levels, creating a grid with *p^k^* points in which evaluations take place. Each perturbation of an input parameter *x_i_* creates a parameter elementary effect (EE) computed as $EE_{i}=\frac{y(x_{1},\ldots ,x_{i}+{△}_{i},\ldots ,x_{k})-y(x)}{{△}_{i}}$, where y(x) is the application output before the perturbation. In our case, the output refers to the metric of interest calculated by comparing the mask generated by the segmentation method to a reference mask. The reference mask is generated using the default input parameters of the image analysis workflow. To account for global SA, we use ${△}_{i}=\frac{p}{2(p-1)}$ that leads to steps slightly larger than half of the input range for input parameters scaled between 0 and 1. The mean (μ), modified mean (μ*—mean of absolute EE values) and standard deviation (σ) of EE are computed for each input parameter (Campolongo *et al.*, 2007). The mean and modified mean represent the effects of an input parameter on the image analysis output, whereas the standard deviation reveals nonlinear effects. MOAT requires n=r(k+1) application runs (or evaluations in the parameter space), with the typical value of *r* being in the range of 5 to 15 (Iooss and Lemaitre, 2015).

### 2.2 Methods to compute importance measures

These methods calculate correlation coefficients between input parameters and application output or between pairs of input parameters. The coefficients implemented in our framework include Pearson’s correlation coefficient (CC), partial correlation coefficient (PCC), Spearman’s rank correlation coefficient (RCC) and partial rank correlation coefficient (PRCC) (Saltelli *et al.*, 2004). The simple and partial correlation coefficients are similar, but the latter excludes effects from other input parameters. The Spearman’s differs from the Pearson’s because the first uses ranked results. When input parameters are orthogonal, the simple and partial correlations are the same. The ranked correlations are helpful when relationships between parameters are non-linear (Saltelli *et al.*, 2004).

The CC for *x* and *y* is calculated as: $Corr(x,y)=r_{xy}=\frac{\sum_{i}\left(x_{i}-\bar{x}\right)(y_{i}-\bar{y})}{\sqrt{\sum_{i}{\left(x_{i}-\bar{x}\right)}^{2}\sum_{i}{\left(y_{i}-\bar{y}\right)}^{2}}}$, where *x* and *y* are two parameters or a parameter and analysis results. The computed value is a comparison metric value as in the screening step. Points evaluated in the input parameter space are selected from a probabilistic exploration. The framework supports the commonly used Monte Carlo sampling, Latin hypercube sampling (LHS) (McKay and Beckman, 1979), quasi-Monte Carlo sampling with Halton or Hammersley sequences, and a few other stochastic methods.

Variance-based Decomposition (VBD) sensitivity method (Weirs *et al.*, 2012) is also available in our framework. VBD splits output uncertainty effects among individual parameters and can account for non-linear relationships among them. VBD computes the ‘main effect’ sensitivity index *S_i_* (Sobol, 2001) and the ‘total effects’ sensitivity index $S_{T_{i}}$ (Saltelli, 2002). The *S_i_* measures the amount of variance in results that can be attributed to parameter *i* alone (first-order effects). If the sum of the *S_i_* values is close to 1.0, most of the output variance is explained by single parameter effects. The total effect index $S_{T_{i}}$ measures the first-order and higher-order effects due to the interaction of parameter *i* with the other parameters. For *k* input parameters and *n* samples, VBD requires n(k+2) application runs, and n can be in the order of thousands (Weirs *et al.*, 2012). It is, thus, important to remove non-influential parameters before VBD is applied.

### 2.3 Parameter auto-tuning algorithms

We have incorporated several parameter optimization methods in our framework and evaluated them experimentally. The methods include Nelder-Mead simplex (NM), Parallel Rank Order (PRO) (Tabatabaee *et al.*, 2005), Genetic Algorithm (GA) (Sareni and Krähenbühl, 1998), GLCCLUSTER, GLCSOLVE (Jones, 2001) and a Bayesian optimization algorithm (Snoek *et al.*, 2012).

The NM method uses a simplex of *k* + 1 vertices in a k-dimensional search space. The PRO is a variant of the NM method that evaluates multiple points in the space concurrently. The Genetic Algorithm (GA) maps each parameter to a gene of an individual. The initial population is created randomly and evolved using crossover and mutation. The crossover uses a one-point crossover between pairs of individuals with a probability of *C*. The mutation in each gene of occurs with an independent probability of *M*. The new population is evaluated and the results are input back to the GA to build another generation. The process continues until a preset number of generations (iterations) is reached. In our experiments, the probabilities *C* and *M* were empirically selected as 0.5 and 0.3, respectively, to maximize performance. We experimentally observed that adding the application default parameters to the initial population did not lead to improvements for our two use-case workflows.

The GLCCLUSTER and DIRECT methods are implemented in the TOMLAB optimization toolkit (http://tomopt.com) and provide a MATLAB interface. We implemented helper codes that wrap these methods. The helper codes are called by the parameter optimization methods with the set of parameter values (i.e. points in the parameter space) to be evaluated. The helper codes in turn invoke our framework’s runtime system to evaluate the points. A recent study has compared several optimization algorithms and showed that GLCCLUSTER obtains very good results (Rios and Sahinidis, 2013), in particular when the number of function evaluations to be performed is small. The Bayesian optimization algorithm (Snoek *et al.*, 2012) is available from the Spearmint software (https://github.com/HIPS/Spearmint). This optimization algorithm builds and explores a probabilistic model of the function being tuned to select points in the search space to be evaluated. The search decision does not rely on local gradients or approximations only. This can benefit optimization of the parameters of complex functions. However, the cost of computing the next set of points to be evaluated can be very high with this method.

### 2.4 Efficient and scalable execution on parallel machines

We have implemented the sensitivity analysis and parameter auto-tuning processes as well as the analysis pipelines used in this paper in the Region Templates (RT) runtime system (Teodoro *et al.*, 2014) for execution on HPC systems with co-processors. The RT runtime system schedules application operations across multiple computation nodes and on co-processors (such as Intel Xeon Phi). The processing structure of a region template application is expressed as a hierarchical dataflow graph. That is, an operation itself can be composed of lower-level operations organized into another dataflow. Application workflows are decomposed into operations that consume, transform and produce region template data objects (also referred to as data regions) instead of reading/writing data directly from/to other tasks or disk. In this way, the application itself does not have to deal with communication of data structures across an HPC platform. The region template data abstraction provides generic data region templates for common data structures, such as pixels, points, arrays (e.g. images or 3D volumes), segmented and annotated objects and regions, which are defined in a spatial and temporal domain. We refer the reader to our earlier publication for more details (Teodoro *et al.*, 2014). The next sections describe two new optimizations in the RT system that address the requirements of the sensitivity study and parameter auto-tuning processes.

#### 2.4.1 Storage management and optimizations

The hierarchical data storage (HDS) is a distributed data management infrastructure with an arbitrary number of storage levels within a node and across a distributed memory machine. The storage hierarchy is defined in a configuration file that includes the number of storage levels, the position of each level in the hierarchy and the level description: type of storage devices (e.g. RAM, SSD, HDD, etc.), capacity, etc. The HDS stores and retrieves data regions produced or requested by an application operation. Output data regions are stored automatically by the runtime system, which iterates through data regions of an operation and stores those marked as output in the highest (i.e. the fastest) level of storage with enough capacity. When a storage level is full, a cache replacement strategy selects data regions to be moved to a lower storage level. Supported data replacement policies are: First-In, First-Out (FIFO) and Least Recently Used (LRU).

We have developed a data locality-aware scheduling (DLAS) that considers the location of data when mapping analysis workflow stages to computing nodes. When an analysis operation finishes, the runtime takes into account the locality of the data produced by that operation to determine the node in which analysis operations that consume the data should be executed. DLAS calculates the amount of data reuse by those operations and inserts the operations into a queue of preferred operations for the node where the data region was produced. A queue of preferred operations is maintained for each node in decreasing order of the amount data reuse. When a node requests work, the runtime system assigns the operation with the maximum data reuse to that node.

#### 2.4.2 Optimized simultaneous parameter evaluation (SPE)

**Fig. 2.**
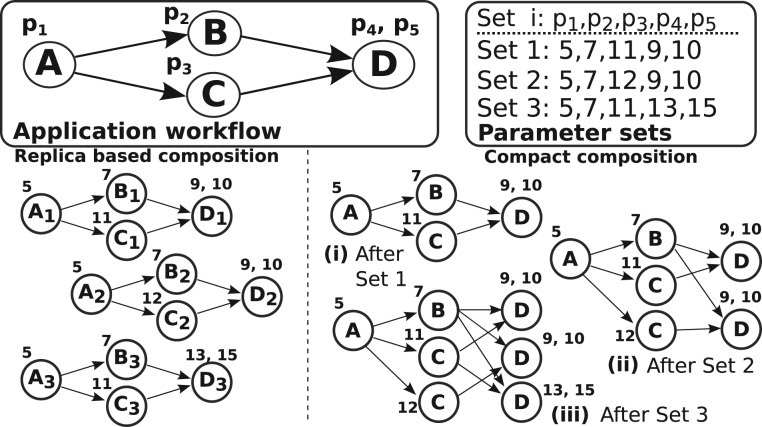
Evaluation of multiple parameter sets. The replica based composition scheme executes independent instances of the same workflow, whereas the compact composition scheme merges the multiple instances to eliminate duplicate computations and data storage

This optimization exploits the evaluation of multiple parameter sets in sensitivity analysis and auto-tuning studies per iteration of each process (Fig. 1). We have developed a strategy that merges and eliminates replicas of common computation paths, when multiple parameter sets are executed. Figure 2 shows two schemes for instantiating an application workflow in this scenario. The *replica based scheme* instantiates and executes the entire application workflow for each parameter set used. The *compact composition scheme* merges the instances of an application workflow into a single, compact workflow graph to reuse common steps in the separate workflow instances. Two steps are common if they use the same input data and parameter values.

The compact workflow graph representation draws from a data structure called FP-Tree (Han *et al.*, 2000). The FP-tree represents sets of transactions in a structure in which common parts of transactions are expressed in a single path on the structure. In our case, we want to merge multiple workflows to create another workflow in which common computations from multiple parameters are eliminated. The complete algorithm to build the compact representation is provided in the Supplementary Section S2. In summary, for each parameter set to be executed, instead of creating a new independent workflow, it searches in the existing workflows for common computation paths. If a common computation path is found, it is instantiated once and its output is reused by the common path to downstream operations that will consume the data.

## 3 Results

We have evaluated the parameter study framework using Glioblastoma brain tumor tissue images obtained from The Cancer Genome Atlas repository for use in brain cancer studies (Kong *et al.*, 2013). We divided the images into tiles, both because a WSI may not fit in memory and in order to distribute the processing of the WSI across multiple nodes and processors (multiple tiles are processed concurrently during the execution of an analysis workflow). We used two analysis workflows, which were made up of normalization, segmentation and comparison steps, for experimental evaluation. The first workflow employed a Watershed based segmentation step with 15 input parameters (Kong *et al.*, 2013), while the second one used a level set method and 7 input parameters (Gao *et al.*, 2016). Please see Table 1 for the list of parameters for each workflow. The experiments were conducted on up to 256 nodes of a distributed memory machine called Stampede (https://portal.xsede.org/tacc-stampede), which is one of the machines supported by the XSEDE consortium (https://www.xsede.org). Each node of the cluster has a dual socket Intel Xeon E5-2680 processors, an Intel Xeon Phi SE10P co-processor and 32 GB RAM. The nodes are inter-connected via Mellanox FDR Infiniband switches.

### 3.1 Sensitivity analysis

We first employed MOAT to identify and filter out non-influential parameters, before using more costly methods, which include correlation coefficients and VBD. This first phase is referred to here as the MOAT phase. The watershed and level set based workflows processed, respectively, 55 WSIs (4276 4K × 4K image tiles) and 1 WSI (71 4K × 4K tissue image tiles) in the experiments—the tile sets did not have any background tiles with no tissue. A smaller dataset is used with the level set workflow because it uses a much more computationally expensive segmentation approach.

#### 3.1.1 Finding important parameters with MOAT

The comparison metric in the experiments was computed as follows. An exclusive-or (XOR) operation was performed between the binary mask generated by the parameters selected by the framework and the binary mask generated by the default parameters of the workflow. The number of pixels with value 1 in the resulting binary mask was counted and used as the value of the comparison metric. We used a parameter space partition with 20 levels for each of the *k* parameters in Table 1, except for those that describe a propagation neighborhood and accept 2 input values only. The number of runs was calculated as n=r(k+1), for the values of *r* ranging from 5 to 15. The experiments used 128 nodes of the Stampede cluster. The total execution times were 15 681s and 6825s, respectively, for the watershed and level set workflows when *r* was 15.

The results for the watershed workflow are presented in Table 2 in which parameters 6, 7, 8 and 14 are shown the most relevant; they have higher μ* and σ values (at least one component higher than 10^9^). Most of the parameters have non-linear interactions, because of the high values of σ. Therefore, we decided to prune the list of parameters conservatively during the MOAT phase. We selected parameters *T*2, *MaxSize*, *MinSizePl*, *MinSizeSeg* in addition to *G*1, *G*2, *Recon* and *MinSize* for the more detailed and costly second phase. These parameters had at least one component (μ* or σ) higher than 10^8^. The other ones (the rows in color red) were discarded from further analysis.

**Table 2:** MOAT analysis for the watershed workflow with r values of 5, 10 and 15

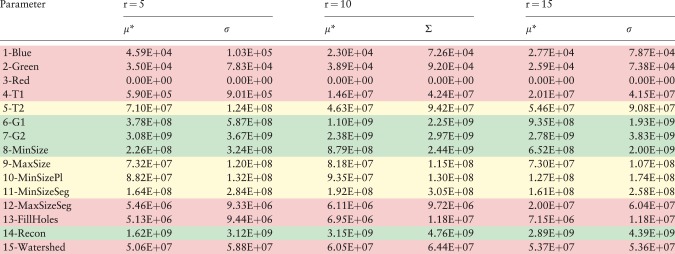

We classify in green, yellow and red, respectively, those parameters having high, medium and low effect on the output. (Color version of this table is available at *Bioinformatics* online.)

The MOAT phase of the level set workflow (shown in Supplementary Table S1 in the supplementary material because of space limitations) included a dummy parameter. This parameter was not passed to the analysis workflow as input. It was used in the experiments to quantify differences in the segmentation output due to the stochastic nature of this analysis workflow. The de-clumping phase of the level-set based segmentation approach is implemented using a randomized clustering strategy and, as a consequence, segmentation results from two runs with the same input parameter values may differ. The experimental results showed that variations in the segmentation output due to the stochastic nature of the segmentation approach were smaller than variations due to the other input parameters.

The OTSU ratio stood out as the most significant parameter for this workflow. At the end of the MOAT phase, all the parameters were selected for further analysis, because at least one component (μ* or σ) of each parameter was above 10^8^.

#### 3.1.2 Importance measures

*Pearson’s and Spearman’s Correlation Coefficients.*

We computed the CC, PCC, RCC and PRCC coefficients by executing each workflow 400 times with the same dataset with different input parameter values. The runs took 27 078s and 22 696s, respectively, for the watershed and level set workflows on 128 computing nodes. The correlation coefficients between the input parameters and the analysis results are presented in Supplementary Table S2 in the supplementary material for both workflows.

For the watershed workflow, the CC values of most parameters are small (about 0.1), with the exception of *G*2 (CC = 0.48). The differences between the CC and PCC values are the evidence of inter parameter correlation effects. These effects are higher for *G*1 and agree with the findings in the MOAT phase. The RCC values of some parameters are higher than their CC values, indicating that those parameters have a monotonic, but not a linear, correlation. This explains why some parameters assumed small CC values in the experiments. The ranking of the parameters, for instance, using RCC is similar to the ranking observed with MOAT. This confirms the MOAT results, but does not facilitate an additional parameter pruning opportunity before VBD is executed.

In the level set workflow, the OTSU was highlighted again as the most important parameter. Its CC and PCC values were almost the same, indicating its effects are orthogonal to those of the other parameters. Although only OTSU appears to be important when the CC is considered, the PCC values showed that the effect of Curvature Weight (CW) increases significantly, after excluding effects from the other parameters. The ranked correlation values (RCC and PRCC) also were higher for OTSU and CW. The same trend was observed between the simple and partial correlations. The *MaxSize* parameter had low correlation values and was excluded from further analysis.

*Variance-Based Decomposition (VBD).* The Sobol’s indices are presented in Table 3 for *k* = 8 and *k* = 5 parameters (for the watershed and level set workflows, respectively) that were not filtered out in the MOAT and Correlation Coefficients phases. We used Saltteli’s approach (Saltelli, 2002) with Monte Carlo sampling. Each experiment required *N *=* n*(*k* + 2) runs. Because of the high computation costs, we limited the value of *n* to 200—this was sufficient because of small variations in the *S_i_* indices as n increased from 100 to 200. The experiments with *n* = 200 and the watershed workflow required 2000 analysis runs with 55 WSIs. They took 150 890 s on 128 nodes, during which 820 TB of data were produced and consumed. The execution with the level set took 211 912 s on 128 nodes.

**Table 3:** VBD results (Main (*S_i_*) and Total ($S_{T_{i}}$) effects)

(a) Results for the watershed based segmentation workflow						
Parameters	n = 50		n = 100		n = 200	
	*S_i_*	$S_{T_{i}}$	*S_i_*	$S_{T_{i}}$	*S_i_*	$S_{T_{i}}$
T2	−1.25e-05	1.32e-07	2.86e-05	6.36e-08	1.67e-03	2.81e-04
G1	3.52e-02	7.57e-02	−1.88e-03	1.44e-01	5.95e-02	9.07e-02
G2	7.80e-01	9.46e-01	5.28e-01	7.57e-01	5.39e-01	8.67e-01
MinSize	1.73e-02	3.92e-02	1.67e-02	4.13e-02	1.34e-02	1.58e-02
MaxSize	4.76e-03	2.80e-04	1.65e-03	1.70e-03	1.29e-04	5.39e-04
MinSizePl	−5.48e-04	4.80e-02	2.31e-02	2.67e-02	1.39e-02	1.99e-02
MinSizeSeg	1.69e-01	1.95e-01	1.38e-01	1.08e-01	8.99e-02	9.37e-02
Recon	−2.24e-02	2.22e-01	−2.70e-02	3.21e-01	2.16e-02	2.06e-01
Sum	1.0		0.73		0.74	
(b) Results for the level set based segmentation workflow						
OTSU	8.91e-01	8.97e-01	9.23e-01	9.42e-01	9.25e-01	9.32e-01
CW	7.33e-02	7.53e-02	1.05e-02	1.48e-02	5.31e-02	5.51e-02
MinSize	1.29e-03	2.84e-03	1.84e-03	2.61e-03	9.51e-04	9.46e-04
MsKernel	3.15e-02	2.56e-02	3.09e-02	3.11e-02	1.71e-02	1.95e-02
LevelSetIt	4.88e-03	5.05e-03	1.03e-03	1.05e-03	2.90e-03	2.12e-04
Sum	1.0		0.96		0.99	

The results in Table 3 for the watershed workflow show that *G*2 has substantially more impact on output variability than the other parameters do. The sum of the *S_i_* indices (0.74 with *n* = 200) is considerably smaller than 1.0, making this a non-additive model. Thus, a large fraction of the output variance cannot be attributed to a single input parameter for this workflow. The higher-order effects ($S_{T_{i}}-S_{i}$) due to parameter interactions are important and cannot be ignored even if *S_i_* is small. This is the case with *Recon* that has a large $S_{T_{i}}$ value and a small *S_i_*. The parameters with higher effect values (*G*1, *G*2, *Recon*) are used in the candidate object identification sub-step of the watershed workflow, highlighting the importance of this sub-step to the overall analysis results.

The sum of main effects is very close to 1.0 for the level set workflow; hence, the model is considered additive. The OTSU parameter explains alone most of the variability in the analysis results (*S_i _*=_* *_0.92 with *n* = 200). This parameter is also used in the candidate object identification substep in the level set workflow. The second most important parameter is the CW parameter, which is used to adapt the smoothness of the boundaries of nuclei. Our experiments show that the other parameters are less important. To validate our findings, we created a panel of segmentation results by varying the values of the two most important parameters and the value of the least important parameter for each segmentation workflow. The panel (see Supplementary Fig. S2 in the supplementary material) shows that the amount of variation in the analysis output agrees with the VBD values computed in the experiments.

In Table 4 we present a summary of the execution times of the sensitivity analysis methods. Even though the number of parameters is pruned through the MOAT, Correlation Coefficients and VBD phases, the execution times increase. This is because the per parameter sample size grows more rapidly than reduction in the set of parameters due to pruning. In the VBD analysis of the watershed workflow, for instance, about half of the original parameters are used, but the execution time of VBD is about 10× higher than MOAT because of the larger sample size. The execution time of MOAT could be used to estimate the execution times of the other more expensive phases by using difference in the sample sizes as a reference. This simple approach, however, tends to be less accurate when the execution time of an analysis workflow varies significantly when input parameters are changed. This is the case with the level set workflow. The ratio of sample sizes of any two phases (MOAT, Correlation Coefficients and VBD) does not reflect accurately variation in the execution time. This is less so with the watershed workflow, because the execution time of this workflow is less sensitive to the input parameters.

**Table 4:** Sensitivity analysis execution times (secs) using 128 computing nodes

Application	Method (Sample Size)		
	MOAT (240)	Importance Measures (400)	VBD (2000)
Watershed	15 681	27 078	150 890
Level Set	6,825	22 696	211 912

### 3.2 Parameter auto-tuning

This section evaluates the auto-tuning algorithms with the goal of maximizing the Dice and Jaccard metrics. These experiments were executed using 15 images manually segmented by a pathologist. First, we tuned the workflows for each image individually to evaluate the results generated by the automatically selected parameters. Second, we carried a random cross validation experiment, separating images into the training and test sets. In this experiment, the entire test set was analyzed using a single parameter set for each workflow.

The parameters of the analysis workflows were varied within the value ranges shown in Table 1. The average values of the Dice and Jaccard metrics for all 15 images are presented in Table 5. The results for each image are provided for reference in Table S3 in the supplementary material. The auto-tuning algorithms were configured to perform a maximum of 100 function evaluations. It is also important to notice that the execution time spent by the optimization algorithm to choose the next parameter set to be evaluated varies among algorithms. It was measured to be about 77s for the Spearmint and less than 10ms for other algorithms. Even though the difference is significant, this cost it is amortized in our analysis because the application execution time to evaluate a parameter is far more expensive.

**Table 5:** Results (avg of the metric over the 15 images) using application default parameters and those selected by the tuning algorithms

Workflow	Dice							Jaccard						
	Default	NM	PRO	GA	GLCCLUSTER	DIRECT	Spearmint	Default	NM	PRO	GA	GLCCLUSTER	DIRECT	Spearmint
Watershed	0.71	0.80	0.80	0.80	0.78	**0.81**	0.80	0.57	0.67	0.67	0.67	0.64	**0.68**	**0.68**
Level Set	0.61	0.75	0.74	0.82	0.61	0.75	**0.86**	0.50	0.71	0.65	0.70	0.47	0.63	**0.79**

The best result for each pair segmentation algorithm and metric of interest is highlighted in bold.

**Fig. 3.**
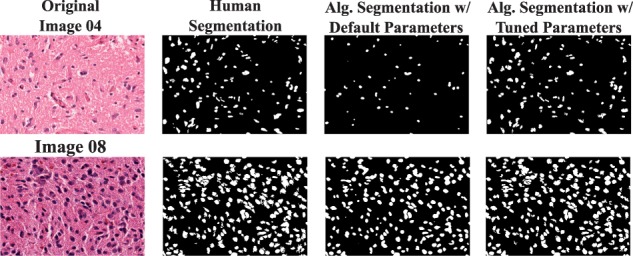
Two image patches are presented with human segmentation and the level set workflow segmentation using default and tuned parameter values. The first image (Image 04) has 0.34 and 0.92 dice values, respectively, with default and tuned parameters. For the second image (Image 08), the dice with default parameter is 0.77 and it is 0.86 after tuning (Color version of this figure is available at *Bioinformatics* online.)

The results presented in Table 5 show that the auto-tuning algorithms significantly improved the quality of the results compared with the results generated by the default input parameters. In the watershed based workflow, the average Dice and Jaccard values were, respectively, 1.14× and 1.19× higher than the values from the default input parameters, whereas they were about 1.41× and 1.58× higher in the level set workflow. Improvements in the Dice and Jaccard values reached 22.2× and 40× depending on the image used, indicating that the results generated by the default input parameters were very poor for some images. Even though the auto-tuning algorithms improved the analysis output quality in most of the cases, an analysis of gains per image shows that no single algorithm is able to produce the best result for all the images in any of the experiments (see Supplementary Table S3 in the supplementary material). The results suggest that an ensemble of tuning algorithms should be used in order to achieve the best results instead of using a single auto-tuning algorithm. A panel with two images is presented in Figure 3 to show the improvement in segmentation output generated by the tuned versus default input parameters.

We also carried out a cross validation experiment in which we used 3 randomly selected images to train the parameter values with the GA method and the remaining 12 images to test the learned parameters. For the watershed workflow, the learned parameters improved the results compared to those from the default parameters on the testing data by 1.10× and 1.13× on average for the Dice and Jaccard metrics, respectively. The standard deviation was smaller than 1%. For the level set based workflow, the results were even better. The average improvement amounts were 1.29× and 1.42×. The results show that the auto-tuning algorithms could significantly improve segmentation results. In addition, the auto-tuning algorithms were able to find a good set of input parameters by examining only up to 100 parameter combinations out of billions possible.

### 3.3 Efficient execution of sensitivity analysis

We experimentally evaluated scalability of our framework as the configuration of hierarchical storage is varied. The experiments included storage configurations with 1 level (1L: file system—FS) and 2 levels (2L: RAM + FS) with FCFS and LRU replacement policies. We also analyzed performance of the data locality-aware coarse-grained scheduling (DLAS) as compared to using the FCFS strategy. A dataset containing 6113 4K × 4K image tiles was used with the watershed based workflow in these analyses because of space constrains. The benefits of the performance optimizations for the level set workflow are similar.

**Fig. 4.**
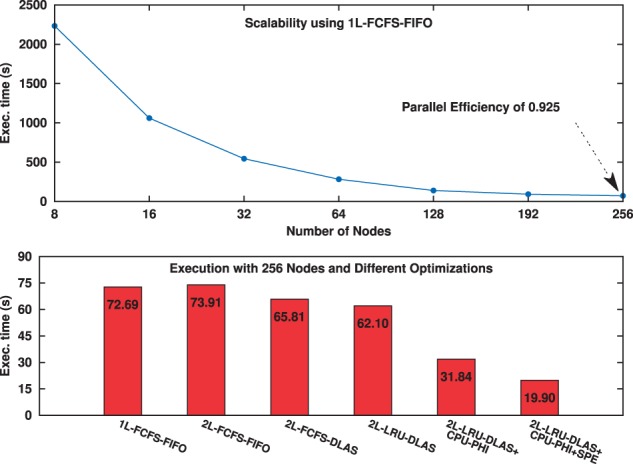
Scalability and performance with different optimizations

The results presented in Figure 4 show that the analysis workflow attained good scalability on a distributed memory machine. The performance of the configuration with a single storage level is faster than the ‘2L FIFO - FCFS’ due to the overhead of maintaining an extra storage level with very low data access hit rate (about 1.5%) in the RAM. However, the ‘2L - FIFO - DLAS’ configuration is better than the single level because of the higher data access hit rate (up to 72%) in the first level storage (RAM) as a result of DLAS scheduling. Moreover, the ‘2L LRU - DLAS’ resulted in the best performance with 1.17× speedup on the 1L configuration due to the improved hit rate (87%). The cooperative use of CPU and Intel Phi improved performance by another 1.95×. The SPE optimization achieved an additional 1.6× speedup.

## 4 Conclusions

Image analysis pipelines are sensitive to input parameters, and more effective application in research of image analysis with large datasets requires better quantification of algorithm sensitivity and tuning of image analysis parameters to produce more accurate and robust results. In this work, we demonstrate that (i) the cost of sensitivity analysis can be reduced by applying a series of optimization phases to identify and filter parameters with small impact on output variability and (ii) the cost of parameter space search can be reduced by intelligently search the space and simultaneously evaluating multiple parameter values on a cluster system while eliminating duplicate computations. We evaluate our approach on a cancer image analysis application using a large-scale cluster system. Our results show that the proposed approach can enable systematic, comparative study of analysis pipelines and improve analysis results when large datasets need to be analyzed. As a future work, we intend to integrate our framework with visual parameter optimization tools and interfaces (Pretorius *et al.*, 2015) for a better visual analysis of results from sensitivity analysis and auto-tuning runs.

## Funding

This work was supported in part by 1U24CA180924-01A1 from the NCI, R01LM011119-01 and R01LM009239 from the NLM, CNPq and NIH K25CA181503. This research used resources of the XSEDE Science Gateways program under grant TG-ASC130023.

*Conflict of Interest*: none declared.

## Supplementary Material

Supplementary Data

## References

[btw749-B1] Campolongo F. et al (2007) An effective screening design for sensitivity analysis of large models. Environ. Modell. Softw., 22, 1509–1518. Modelling, computer-assisted simulations, and mapping of dangerous phenomena for hazard assessment.

[btw749-B2] Dice L.R. (1945) Measures of the amount of ecologic association between species. Ecology, 26, 297–302.

[btw749-B3] Gao Y. et al (2016) Hierarchical nucleus segmentation in digital pathology images. Proc. SPIE, 9791, 979117–979117–6. 10.1117/12.2217029 PMC4927003 27375315

[btw749-B4] Han J. et al (2000) Mining Frequent Patterns Without Candidate Generation. In: *Proc. of the 2000 ACM SIGMOD Int. Conf. on Management of Data*, SIGMOD ’00.

[btw749-B5] Iooss B., Lemaitre P. (2015) A review on global sensitivity analysis methods In Dellino G., Meloni C. (eds.) Uncertainty Management in Simulation-Optimization of Complex Systems, Volume 59 of Operations Research/Computer Science Interfaces Series, pp. 101–122. Springer US.

[btw749-B6] Jones D.R. (2001) Direct Global Optimization Algorithm, *Encyclopedia of Optimization*. pp. 431–440. Springer US, Boston, MA.

[btw749-B7] Kong J. et al (2013) Machine-based morphologic analysis of glioblastoma using whole-slide pathology images uncovers clinically relevant molecular correlates. PLoS ONE, 8, 1–11. 10.1371/journal.pone.0081049 PMC3827469 24236209

[btw749-B8] Kumar S., Hebert M. (2003) Discriminative random fields: a discriminative framework for contextual interaction in classification. In: *Proc. 9th IEEE International Conference on Computer Vision*, pp. 1150–1157.

[btw749-B9] McKay M.D., Beckman R.J. W. J. C. (1979) A comparison of three methods for selecting values of input variables in the analysis of output from a computer code. Technometrics, 21, 239–245.

[btw749-B10] McIntosh C., Hamarneh G. (2007) Is a single energy functional sufficient? Adaptive energy functionals and automatic initialization. In: *Lecture Notes in Computer Science, Medical Image Computing and Computer-Assisted Intervention (MICCAI)*, vol. 4792, pp. 503–510. 10.1007/978-3-540-75759-7_61 18044606

[btw749-B11] Morris M.D. (1991) Factorial sampling plans for preliminary computational experiments. Technometrics, 33, 161–174.

[btw749-B12] Pretorius A. et al (2015) Visual parameter optimisation for biomedical image processing. BMC Bioinformatics, 16, 1–13. 26329538 10.1186/1471-2105-16-S11-S9 PMC4547193

[btw749-B13] Rios L.M., Sahinidis N.V. (2013) Derivative-free optimization: a review of algorithms and comparison of software implementations. J. Global Optim., 56, 1247–1293.

[btw749-B14] Saltelli A. (2002) Making best use of model evaluations to compute sensitivity indices. Comput. Phys. Commun., 145, 280– 297.

[btw749-B15] Saltelli A. et al (2004) Sensitivity Analysis in Practice: A Guide to Assessing Scientific Models. Wiley.

[btw749-B16] Sareni B., Krähenbühl L. (1998) Fitness sharing and niching methods revisited. IEEE Trans. Evol. Comput., 2, 97–106.

[btw749-B17] Schultz T., Kindlmann G.L. (2013) Open-box spectral clustering: applications to medical image analysis. IEEE Trans. Vis. Comput. Graph, 19, 2100–2108. 24051776 10.1109/TVCG.2013.181

[btw749-B18] Snoek J. et al (2012) Practical bayesian optimization of machine learning algorithms In: Pereira F. et al (eds.) Advances in Neural Information Processing Systems 25, pp. 2951–2959. Curran Associates, Inc.

[btw749-B19] Sobol I. (2001) Global sensitivity indices for nonlinear mathematical models and their Monte Carlo estimates. Math. Comput. Simul., 55, 271– 280.

[btw749-B20] Szummer M. et al (2008) Learning CRFs using graph cuts. In; *Proceedings of the 10th European Conference on Computer Vision: Part II*, ECCV ’08, pp. 582–595. Springer-Verlag, Berlin, Heidelberg.

[btw749-B21] Tabatabaee V. et al (2005) Parallel parameter tuning for applications with performance variability. In: *Proc. of the 2005 ACM/IEEE Conf. on Supercomputing.*

[btw749-B22] Teodoro G. et al (2014) Region templates: Data representation and management for high-throughput image analysis. Parallel Comput., 40, 589–610. 26139953 10.1016/j.parco.2014.09.003 PMC4484879

[btw749-B23] Torsney-Weir T. et al (2011) Tuner: principled parameter finding for image segmentation algorithms using visual response surface exploration. IEEE Trans. Vis. Comput. Graph., 17, 1892–1901. 22034306 10.1109/TVCG.2011.248

[btw749-B24] Weirs V.G. et al (2012) Sensitivity analysis techniques applied to a system of hyperbolic conservation laws. Reliab. Eng. Syst. Saf., 107, 157–170.

