# Supplementary material for: Algorithm sensitivity analysis and parameter tuning for tissue image segmentation pipelines
Source: Bioinformatics. 2016 Dec 30;33(7):1064–72. doi: 10.1093/bioinformatics/btw749 (PMC5409344; doi:10.1093/bioinformatics/btw749)
Supplement: Supplementary Data [file btw749_supp.pdf]

Supplementary Materials for Algorithm Sensitivity Analysis and Parameter Tuning for Segmentation Pipelines on Large Whole Slide Tissue Imaging Datasets

George Teodoro[1,3,*], Tahsin M. Kurc[3], Luís F. R. Taveira[1], Alba C. M. A. Melo[1], Yi Gao[3], Jun kong[2] and Joel H. Saltz[31]

[1]Department of Computer Science, University of Brasília, Brazil
[2]Biomedical Informatics Department, Emory University, USA
[3]Biomedical Informatics Department, Stony Brook University, USA
[*]To whom correspondence should be addressed. Tel +55-61-3107-3663; Email: teodoro@unb.br.

October 13, 2016

S1 Use Case Application Workflows

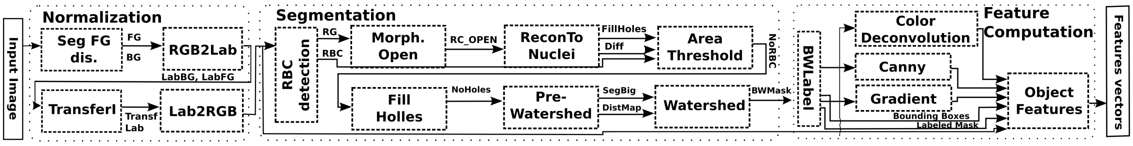

(a) Watershed based segmentation workflow.

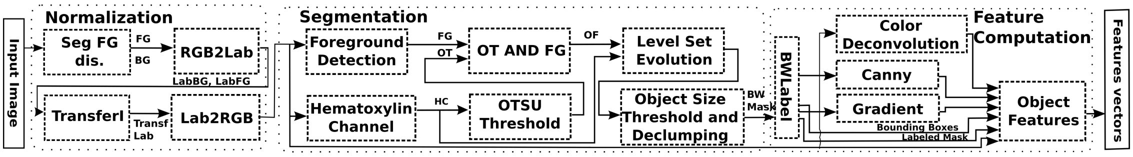

(b) Level Set based segmentation workflow.

Figure S1: Two example analysis workflows with their cascade of internal operations. They have the same high level computation stages: normalization, segmentation and feature computation. The stages of normalization and feature computation are shared among between the workflows, but different techniques are used to implement the segmentation phase. The first (Figure (a)) uses morphological operations along with watershed to delineate and separate clumped cells. The second (Figure (b)) employs the level set strategy and a mean shift based clustering to delineate and separate clumped cells

S2 Simultaneous Parameter Evaluation

This section presents the algorithm to construct the compact representation (Algorithm S1). To simplify the presentation of the algorithm, we assume that both the compact graph (comGraph) and each instance of the application graph (appGraphInst) to be merged have a single start root node. The algorithm takes the application workflow graph (appGraph) and parameter sets to be tested simultaneously as input (parSets) and outputs the compact graph (comGraph). It iterates over each parameter set (lines 3-5) to instantiate a replica of the application workflow graph with parameters from set. It then calls MERGEGRAPH to merge the replica to the compact representation.

Algorithm S1 Compact Graph Construction

```
 1: Input: appGraph; parSets;
 2: Output: comGraph;
 3: for each set ∈ parSets do
 4:     appGraphInst = INSTANTIATEAPPGRAPH(set);
 5:     MERGEGRAPH(appGraphInst.root, comGraph.root);
 6: procedure MERGEGRAPH(appVer, comVer)
 7:     for each v ∈ appVer.children do
 8:         if (v' ← find(v, comVer.children)) then
 9:             MERGEGRAPH(v, v');
10:         else
11:             if ((v' ← PendingVer.find(v))=∅) then
12:                 v' ← clone(v)
13:                 v'.depsSolved ← 1
14:                 comVer.children.add(v')
15:                 if v'.deps ≥ 1 then
16:                     PendingVer.insert(v')
17:                 MERGEGRAPH(v, v');
18:             else
19:                 comVer.children.add(v')
20:                 v'.depsSolved ← v'.depsSolved+1
21:                 if v'.depsSolved = v'.deps then
22:                     PendingVer.remove(v')
23:                 MERGEGRAPH(v, v')
```

The MERGEGRAPH procedure walks simultaneously in an application workflow graph instance and in the compact representation. If a path in the application workflow graph instance is not found in the latter, it is added to the compact graph. The MERGEGRAPH procedure receives the current set of vertices in the application workflow (appVer) and in the compact graph (comVer) as a parameter and, for each child vertex of the appVer, finds a corresponding vertex in the children of comVer. Each vertex in the graph has a property called *deps*, which refers to its number of dependencies. The find step considers the name of a stage and the parameters used by the stage. If a vertex is found, the path already exists, and the same procedure is called recursively to merge sub-graphs starting with the matched vertices (lines 8-9). When a corresponding vertex is not found in the compact graph, there are two cases to be considered (lines 10-23). In the first one, the searched node does not exist in comGraph. The node is created and added to the compact graph (lines 11-17). To check if this is the case, the algorithm verifies if the node (v) has not been already created and added to comGraph as a result of processing another path of the application workflow that leads to v. This occurs for nodes with multiple dependencies, e.g., D in Figure 2. If the path (A,B,D) is first merged to the compact graph, when C is processed, it should not create another instance of D. Instead, the existing one should be added to the children list as the algorithm does in the second case (lines 19-23). The PendingVer data structure is used as a look-up table to store such nodes with multiple dependencies during graph merging.

The proposed algorithm assumes that the application workflows are directed graphs. If a stage is used multiple times in the same application, it is necessary to rename the repeated stage to prevent the algorithm from finding incorrect nodes in the look-up of pending nodes (line 11).

S3 Supplementary Results

Table S1: MOAT analysis for the level set workflow with r values of 5, 10, and 15. We classify in green, yellow, and red, respectively, those parameters having high, medium, and low effect on the output.

Parameter	r = 5		r=10		r=15	
	μ^*	σ	μ^*	σ	μ^*	σ
1-OTSU	9.64e+07	1.35e+08	1.12e+08	1.19e+08	1.05e+08	1.19e+08
2-CW	1.97e+07	3.33e+07	1.47e+07	2.50e+07	1.62e+07	2.70e+07
3-MinSize	5.89e+06	7.42e+06	6.37e+06	7.62e+06	6.06e+06	7.32e+06
4-MaxSize	1.77e+05	3.94e+05	5.24e+05	1.06e+06	1.12e+06	3.15e+06
5-MsKernel	2.96e+07	4.23e+07	3.34e+07	4.43e+07	3.17e+07	4.15e+07
6-LevelSetIt	1.27e+07	2.42e+07	7.22e+06	1.70e+07	7.99e+06	1.67e+07
7-Dummy	1.93e+05	3.48e+05	1.96e+05	3.84e+05	1.81e+05	3.58e+05

Table S2: Pearson's and Spearman's Simple and Partial Correlation Coefficients of the segmentation workflows with respect to changes input parameters and their impact to the output changes. The output changes are measured in terms of the number of pixels modified in the segmentation output as parameter values are changes.

(a) Results for the watershed based segmentation workflow.

Parameter	CC	PCC	RCC	PRCC
T2	-4.94e-02	-9.27e-02	3.97e-02	2.13e-02
G1	1.01e-01	1.57e-01	1.41e-01	1.83e-01
G2	4.83e-01	5.08e-01	3.74e-01	3.99e-01
MinSize	1.02e-01	1.25e-01	1.38e-01	1.36e-01
MaxSize	7.72e-03	4.46e-02	-3.88e-02	-8.21e-03
MinSizePl	8.79e-02	1.91e-02	1.29e-01	7.27e-02
MinSizeSeg	-6.29e-02	-3.31e-02	3.96e-02	7.10e-02
Recon	9.16e-02	1.14e-01	8.35e-02	9.40e-02

(b) Results for the level set based segmentation workflow.

Parameter	CC	PCC	RCC	PRCC
OTSU	7.47e-01	7.52e-01	5.90e-01	6.09e-01
CW	-5.18e-02	-1.05e-01	-1.86e-01	-2.63e-01
MinSize	5.05e-03	-1.88e-02	6.97e-02	9.25e-02
MaxSize	-3.78e-02	5.93e-03	-9.35e-03	2.84e-02
MsKernel	-3.74e-02	9.66e-02	-3.65e-02	4.40e-02
LevelSetIt	-6.50e-02	-7.48e-02	-7.73e-02	-8.01e-02

Table S3: Comparison of results generated using Default application parameters and those selected by the tuning algorithms NM, PRO, GA, GLCLUSTER (GLC), DIRECT (DIR), and Spearmint (SPE).

(a) Results for the watershed based segmentation workflow.

Image	Dice							Jaccard						
	Default	NM	PRO	GA	GLC	DIR	SPE	Default	NM	PRO	GA	GLC	DIR	SPE
1	0.71	**0.81**	**0.81**	0.79	0.71	0.78	0.80	0.55	**0.68**	**0.68**	0.67	0.55	0.64	0.67
2	0.75	0.83	0.81	0.81	0.83	**0.84**	0.82	0.60	0.70	0.68	0.69	0.72	**0.73**	0.71
3	0.76	0.80	**0.81**	0.80	0.81	0.81	**0.81**	0.61	0.67	0.68	0.68	0.67	**0.69**	**0.69**
4	0.71	0.84	0.84	0.85	0.83	**0.86**	0.84	0.55	0.73	0.73	0.73	0.70	**0.75**	0.73
5	0.71	0.76	0.76	0.77	0.76	**0.78**	0.77	0.55	0.62	0.61	0.62	0.62	**0.64**	0.63
6	0.82	**0.85**	0.84	0.84	0.82	0.83	0.84	0.70	**0.73**	**0.73**	**0.73**	0.70	0.71	**0.73**
7	0.71	**0.83**	**0.83**	0.82	0.73	**0.83**	0.82	0.67	**0.71**	0.70	0.70	0.58	**0.71**	0.70
8	0.69	0.79	0.79	0.79	0.74	**0.80**	0.79	0.53	0.65	0.65	0.65	0.58	**0.66**	0.65
9	0.61	0.72	0.72	**0.73**	0.71	0.72	**0.73**	0.44	0.57	0.57	0.57	0.55	0.57	**0.58**
10	0.70	0.78	**0.79**	**0.79**	0.75	0.78	0.78	0.53	0.63	0.65	0.65	0.59	0.66	**0.67**
11	0.57	0.70	0.70	0.72	0.69	**0.74**	0.72	0.40	0.54	0.54	**0.56**	0.52	**0.56**	0.58
12	0.68	0.78	0.79	0.79	0.78	**0.81**	**0.81**	0.52	0.64	0.66	0.65	0.63	0.65	**0.69**
13	0.71	0.83	0.83	0.83	**0.85**	**0.85**	0.82	0.60	0.71	0.71	0.70	0.74	**0.75**	0.70
14	0.79	0.84	0.84	0.84	**0.85**	**0.85**	0.84	0.65	0.73	0.72	0.72	0.74	**0.75**	0.73
15	0.72	**0.86**	**0.86**	0.85	0.85	**0.86**	**0.86**	0.66	**0.76**	0.75	0.75	0.74	0.75	0.75
Sum	10.65	12.02	12.02	12.03	11.71	**12.14**	12.05	8.56	10.07	10.06	10.07	9.63	**10.22**	10.21

(b) Results for the level set based segmentation workflow.

Image	Dice							Jaccard						
	Default	NM	PRO	GA	GLCC	DIR	SPE	Default	NM	PRO	GA	GLCC	DIR	SPE
1	0.40	**0.86**	**0.86**	0.83	0.74	0.84	0.84	0.25	**0.76**	**0.76**	0.70	0.58	0.74	0.74
2	0.10	0.87	**0.88**	0.71	0.31	0.32	0.68	0.05	0.77	0.77	0.54	0.18	0.30	**0.78**
3	0.04	0.88	**0.89**	0.56	0.31	0.32	0.85	0.02	0.78	**0.80**	0.34	0.31	0.19	0.77
4	0.34	**0.92**	0.91	0.85	0.75	0.87	0.91	0.20	**0.85**	0.84	0.59	0.39	0.38	0.82
5	0.19	**0.82**	**0.82**	0.75	0.32	0.36	0.81	0.10	**0.69**	**0.69**	0.61	0.19	0.26	**0.69**
6	0.73	0.89	0.87	0.89	0.88	**0.90**	**0.90**	0.57	0.79	0.79	0.80	0.78	**0.82**	**0.82**
7	0.69	**0.88**	0.87	**0.88**	0.85	0.87	0.87	0.53	**0.78**	0.68	0.77	0.74	**0.78**	**0.78**
8	0.77	**0.86**	**0.86**	**0.86**	0.85	0.85	**0.86**	0.63	**0.76**	**0.76**	0.75	0.75	0.75	0.73
9	0.83	0.86	0.77	**0.87**	0.61	**0.87**	**0.87**	0.71	0.74	0.70	0.77	0.44	0.76	**0.79**
10	0.87	**0.93**	0.85	0.89	0.66	0.88	0.90	0.77	0.76	0.75	0.80	0.49	0.79	**0.81**
11	0.77	0.72	0.77	0.79	0.40	0.79	**0.84**	0.63	0.61	0.58	0.68	0.25	0.65	**0.76**
12	0.84	0.84	0.82	0.85	0.54	0.82	**0.88**	0.72	0.73	0.71	0.73	0.56	0.70	**0.79**
13	0.90	0.21	0.21	**0.91**	0.77	0.87	0.88	**0.82**	0.12	0.12	**0.82**	0.62	0.77	**0.82**
14	0.86	0.40	0.41	**0.88**	0.62	0.86	0.87	0.75	0.76	0.63	**0.78**	0.39	0.75	**0.83**
15	0.89	0.37	0.37	**0.91**	0.61	0.90	**0.91**	0.80	0.83	0.23	0.82	0.44	0.83	**0.88**
Sum	9.22	11.32	11.17	12.42	9.22	11.32	**12.87**	7.56	10.72	9.81	10.52	7.11	9.47	**11.81**

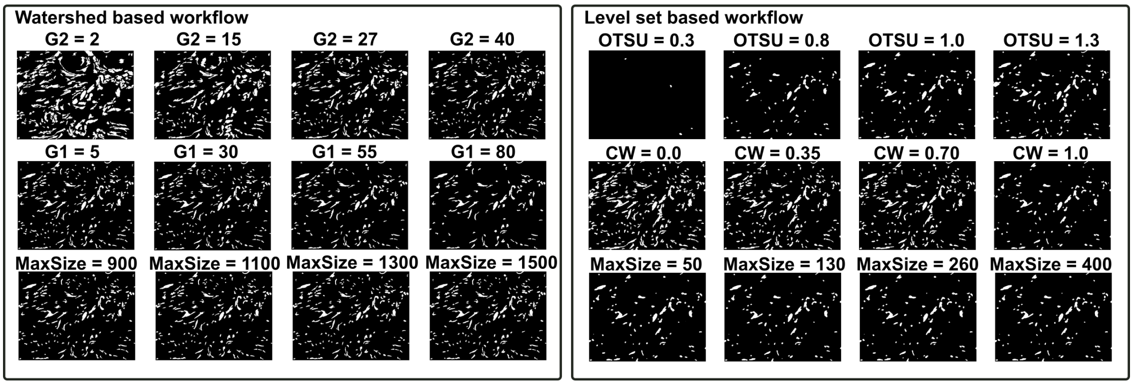

Figure S2: Example segmentation results showing the output impacts as the two most influential and the least important parameter identified by VBD are varied in their range value.
